# Carbohydrate and Glutamine Supplementation Attenuates the Increase in Rating of Perceived Exertion during Intense Exercise in Hypoxia Similar to 4200 m

**DOI:** 10.3390/nu12123797

**Published:** 2020-12-11

**Authors:** Aline V. Caris, Ronaldo V. Thomatieli-Santos

**Affiliations:** 1 Department of Psychobiology, Universidade Federal de São Paulo, São Paulo 04023-062, Brazil; alinecaris@hotmail.com; 2 Department of Bioscience, Universidade Federal de São Paulo, São Paulo 11015-020, Brazil

**Keywords:** high altitude, maltodextrin, fatigue, glucose, glutamine, mood

## Abstract

The rating of perceived exertion (RPE) indicates the feeling of fatigue. However, hypoxia worsens the condition and can worsen RPE. We evaluated whether carbohydrate and glutamine supplementation alters RPE and physiological markers in running at 70% peak oxygen uptake until exhaustion in a simulated altitude of 4500 m. Nine volunteers underwent three running tests at 70% peak oxygen uptake until exhaustion: (1) hypoxia and placebo, (2) hypoxia and 8% maltodextrin, and (3) hypoxia after six days of glutamine supplementation (20 g/day) and 8% maltodextrin. The exercise and supplementation were randomized and double-blinded. Lactate, heart rate, haemoglobin O_2_ saturation (SpO_2_%), and RPE (6–20 scale) were analyzed at the 15th and 30th min. The level of significance was set at *p* ≤ 0.05. SpO_2_% decreased at the 15th and 30th minutes compared to resting in placebo, carbohydrate, and glutamine supplementation. RPE increased at the 30th minute compared to the 15th minute in placebo and carbohydrate supplementation; however, there was no difference in the glutamine supplementation condition. Heart rate and lactate increased after the 15th and 30th minutes compared to resting, similar to the three conditions studied. We conclude that previous supplementation with glutamine and carbohydrate during intense exercise in hypoxia similar to 4500 m can attenuate the increase in RPE by the increase in glycemia and can be a useful strategy for people who exercise in these conditions.

## 1. Introduction

The rating of perceived exertion (RPE) is a subjective scale widely used to derive the intensity of exercise and the total effort spent in an exercise session. Additionally, it has a high correlation with heart rate and lactate concentration during exercise [1]. Some studies at sea level suggest that RPE indicates tiredness or weakness, since it is a psychophysiological scale useful for measuring fatigue during exercise [2].

High altitude regions, characterized by hypoxia due to reduced barometric pressure and O_2_ pressure, significantly impact cognitive function [3,4]. Dimensions of mood, such as vigor, fatigue, and anger, worsen in hypoxia due to high altitudes, decreasing the recognition of perceptions, representations, and concepts, in addition to increasing depression, tension, and confusion [5,6]. The effects of hypoxia depend on the intensity and duration of exposure. Exposures above 2500 m can worsen mood and cause cognitive impairment [7]. It has been shown that tension, fatigue, and stamina progressively worsen at altitudes above 6000 m, even if exposure is for a short time, such as less than 60 min [8].

Many people exercise in hypoxic conditions, for leisure, tourism, mountain climbing, football games, or even because they live in high altitude regions [9]. However, few studies have evaluated RPE during exercise in hypoxia. Mellor et al. showed the correlation between symptoms of acute mountain sickness and trekking at different altitudes from 3833 m [10]. Another study showed that during steady-state exercise, RPE increased at 4300 m compared to 3000 m, suggesting a possible effect of hypoxia [11]. The increase in RPE seems to correlate with the reduction in SaO_2_ and increase in heart rate at altitudes greater than 2543 m [12]. Psychobiological changes over 2500 m, including mood and congestion, may worsen the self-reported perception of effort [13].

A previous study showed that carbohydrate supplementation reduced RPE during moderate exercise (50% peak oxygen uptake) and RPE area under curve for 60 min in hypoxia equivalent to 4200 m, while the heart rate/RPE ratio increased, suggesting lower RPE [13]. However, the effects of intense exercise on RPE in hypoxia are not unquestionably known.

Carbohydrate and glutamine supplementation is associated with partial recovery of SaO_2_% after exercise in hypoxia [14,15], which raises the question of whether supplementation could mitigate the effects of hypoxia on RPE mediated by improved SaO_2_%.

Studies show that small decreases in blood glucose can negatively influence mood indicators, including irritability, mental alertness, anxiety, and fatigue [16]. At the same time, the accumulation of lactate may be associated with worsening cognitive performance [17]. Moreover, carbohydrate intake and increase in circulating levels of glucose and carbohydrate oxidation tend to attenuate RPE and promote a positive impact on cognitive behavior [18,19], since the brain depends on glucose as fuel [16]. Oral ingestion of glutamine can exert central effects because it is the substrate for the synthesis of two major neurotransmitters in the central nervous system, including glutamate, thereby modulating motor, cognitive, and behavioral responses [20,21].

It is essential to assess whether carbohydrate and glutamine supplementation influences RPE and physiological markers during exercise in hypoxia and mood. This study evaluated the importance of supplementing with carbohydrates and glutamine over RPE and physiological indicators during exercise at 70% peak oxygen uptake until exhaustion in hypoxia equivalent to 4500 m.

## 2. Methods

### 2.1. Participants 

The sample size was calculated by G * Power version 3.1 statistical program, with explanatory power (Power) of 0.80 and an α level equal to 0.05 was considered. There was a need for a sample of 10 volunteers. Though the sample of 10 volunteers was needed, the study was conducted with nine healthy and trained male volunteers. The volunteers presented 26.4 ± 3.5 years, 76.8 ± 8.7 kg, 1.75 ± 0.14 m, 25.0 ± 2.3 kg/m^2^, maximum oxygen consumption of 50.7 ± 5.6 mL/kg/min, and maximum heart rate of 191.8 ± 9.2 beats × min^−1^. The study procedures were approved by the Ethics Committee of the Federal University of São Paulo (UNIFESP)-(CEP-0620/09) and are under the guidelines established in the Helsinki International Declaration (1964). 

### 2.2. Experimental Design

The volunteers visited the laboratory on 4 days. On the first day, the volunteers received information on how the study would be carried out, including the objectives and procedures to which they would be submitted. After agreeing to participate, the volunteers signed the Free and Informed Consent Form, realizing an electrocardiogram and spirometry. In the following three weeks, the volunteers were supplemented and performed the exercise. Supplementation was randomized and blinded under the following conditions:

(I) Hypoxia placebo for glutamine (for 6 days) and placebo for maltodextrin (H);
(II) Hypoxia carbohydrate with 8% maltodextrin (200 mL/every 20 min during) and placebo for glutamine (HC);
(III) Hypoxia, carbohydrate and glutamine supplementation (20 g/day for six days), supplemented with 8% maltodextrin (200 mL/every 20 min) (HCG).

### 2.3. Supplementation

The volunteers drank 200 mL of 8% maltodextrin (Probiotic^®^ Laboratories, Embu das Artes, São Paulo, Brazil) or placebo (Crystal Light^®^, Kraft Foods, Northfield, IL, USA) every 20 min during exercise. Regarding glutamine supplementation during the six days before the test, the volunteers consumed 20 g/day of glutamine (Probiotic^®^ Laboratories, Embu das Artes, São Paulo, Brazil) or placebo (corn starch 10 g + lactose 10 g). The dose of maltodextrin complies with the American College of Sports Medicine guidelines for sports nutrition [22], while the glutamine supplementation used was a dose similar to one used for patients with serious illnesses that may reduce infections, hospital length of stay, and mortality [23]. Placebo, maltodextrin, and glutamine had the same color, smell, and taste and were ingested at night before sleeping. After the study, the volunteers asked what they had consumed. No volunteer has set the correct randomization order.

### 2.4. Simulation of Hypoxia 

For hypoxia induction, an altitude simulator (CAT—Colorado Altitude Training ™/CAT-12^®^ Air Unit) composed of a normobaric chamber capable of simulating up to 4500 m of altitude was used. Hypoxia at this altitude is equivalent to a barometric pressure of 433 mmHg and an inspired oxygen fraction of 13.5% O_2_).

### 2.5. Determination of Peak Oxygen Uptake

The test was performed on a treadmill with progressive intensity and an inclination of 1%. The first stage was at a speed of 7.0 km/h. Every minute, speed was increased by 1.0 km/h until the volunteers were exhausted. Heart rate was measured every minute with a frequency meter, blood pressure was monitored using a sphygmomanometer and stethoscope, and the perception of effort was assessed using the RPE scale (6–20). The equipment was calibrated according to the manufacturer’s recommendations to measure ventilatory parameters

### 2.6. Physical Exercise 

The volunteers remained at rest for 2 h in the chamber before beginning the exercise. The exercises were performed over 3 consecutive weeks, and the interval between each exercise was 6 days. The exercises were performed after an overnight fast to prevent the influence of diet on exercise and the evaluated parameters. Tests started at 7:30 a.m. to avoid circadian influences.

### 2.7. Performance Evaluation 

The volunteers ran at 70% peak oxygen in hypoxia until voluntary exhaustion, that is, the inability to maintain speed for 15 consecutive seconds or until the moment when the volunteers interrupted the exercise of their own will due to fatigue, even after being encouraged to continue.

### 2.8. Rating of Perceived Exertion (RPE)

RPE was assessed at the 15th and 30th minutes using a subjective scale of perceived exertion, ranging from 6 to 20. Before the exercises, the researcher showed the RPE scale, explained the meaning of the scale, and answered questions so that the volunteers were familiar with the variations of 6 to 20 arbitrary units.

### 2.9. Heart Rate (HR)

Heart rate was assayed at rest, on the 15th and 30th minutes of exercise by the frequency meter (Polar^®^, Advantage Model NV, Kempele, Finland).

### 2.10. Lactate and Glucose

Blood lactate and glucose were evaluated at rest, on the 15th and 30th minutes of the exercise. In the three moments, 25 μL of blood from the earlobe was collected and stored in tubes containing 50 μL of 1% NaF. All samples were stored in a freezer at −80 °C for proper conservation and further analysis. A SPORT YSI 2300 lactate electro-enzyme analyzer (Yellow Springs Inc.^®^, Yellow Springs, OH, USA) was used to measure the glucose and lactate concentrations of the samples.

### 2.11. Haemoglobin O_2_ Saturation (SpO_2_)

The SpO_2_ was monitored with a finger oximeter (FingerPulse^®^ model MD300C202, Plymouth, MN, USA) before exercise and every 15 min during exercise.

### 2.12. Statistical Analysis

The Shapiro–Wilk test verified the normality of results. The mean ± standard deviation was used for descriptive analysis. To verify the interactions between condition and time, two-way ANOVA and Tukey’s test were used. A significance level of *p* ≤ 0.05 was adopted. Statistica^®^ 7.0 software (StatSoft, Inc., Tulsa, OK, USA) was used to perform the comparisons.

## 3. Results

**Figure 1:**
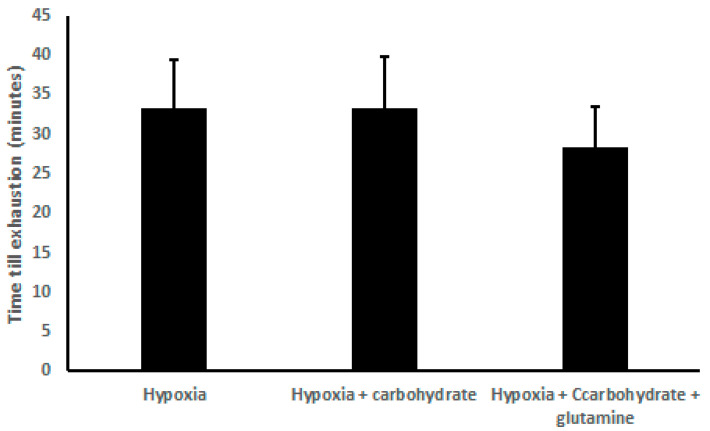
Results of time exhaustion (min), demonstrated as mean ± standard deviation. Two-way ANOVA and Tukey’s test were used, *n* = 9 volunteers. For *p* < 0.05.

There was no difference in the exhaustion time, comparing H (33.33 ± 6.00 min), HC (33.33 ± 6.50 min), and HCG (28.33 ± 5.27 min) (Figure 1). 

**Figure 2:**
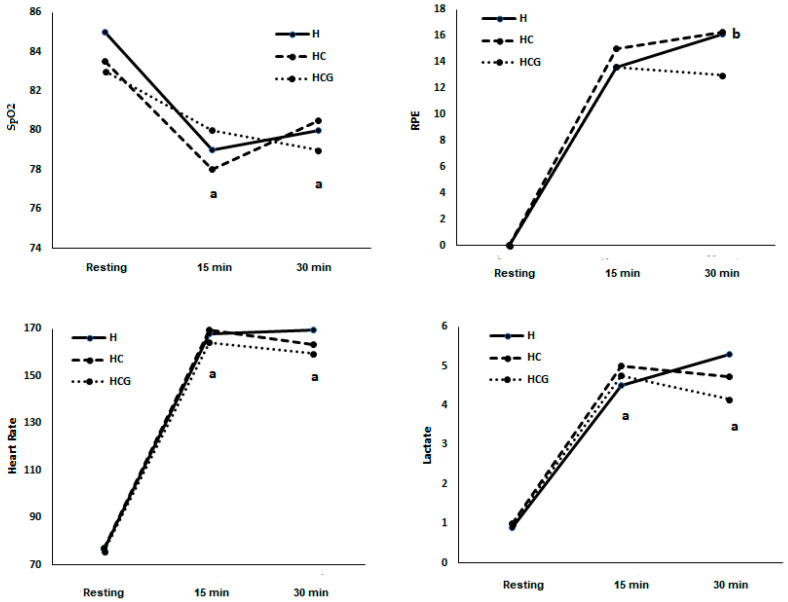
SpO_2_ (%), rating of perceived exertion (RPE) (scale 6–20), heart rate (beats × min^−1^), and lactate (mmol) demonstrated as a mean. Two-way ANOVA and Tukey’s test were used, *n* = 9 volunteers. ^a^ was different from the resting condition. ^b^ was different from the time 15’. For *p* < 0.05. Hypoxia (H), hypoxia + carbohydrate (HC), and hypoxia + carbohydrate + glutamine (HCG).

In condition H, SpO_2_% decreased at the 15th minute compared to resting (78.89 ± 1.08) and the 30th minute compared to resting (80.67 ± 1.44 vs. 85.22 ± 1.54). In the HC condition, SpO_2_% decreased at the 15th minute (78.67 ± 1.33 vs. 83.78 ± 1.01) compared to resting and the 30th minute compared to resting (80.20 ± 2.17 vs. 83.78 ± 1.01). In the HCG condition, SpO_2_% decreased at the 15th minute (80.11 ± 1.05 vs. 83.67 ± 0.83) compared to resting and the 30th minute compared to resting (79.80 ± 1.09 vs. 83.67 ± 0.83) (Figure 2). There was no statistical difference between the three conditions and between the 15th and 30th minutes in the three conditions (Figure 2).

In condition H, RPE increased at the 30th minute compared to the 15th minute (13.67 ± 0.86 vs. 16.14 ± 1.23). There was no statistical difference between the three conditions and between the 15th and 30th minutes in the three conditions (Figure 2).

In condition H, HR increased at the 15th (168.00 ± 4.30) and 30th (169.83 ± 5.03) minutes compared to resting (76.78 ± 2.47). In the HC condition, HR increased at the 15th (169.44 ± 5.43) and 30th (163.20 ± 5.22) minutes compared to resting (77.11 ± 2.62). In the HCG condition, HR increased at the 15th (164.11 ± 6.14) and 30th (159.40 ± 5.04) minutes compared to resting (75.67 ± 1.69). There was no statistical difference between the three conditions (Figure 2). There was no statistical difference between the three conditions and between the 15th and 30th minutes in the three conditions (Figure 2).

In condition H, lactate increased at the 15th (4.55 ± 0.71) and 30th (5.37 ± 1.39) minutes compared to resting (0.97 ± 0.10). In the HC condition, lactate increased at the 15th (5.01 ± 0.65) and 30th (4.72 ± 0.75) minutes compared to resting (0.98 ± 0.08). In the HCG condition, lactate increased at the 15th (4.73 ± 0.90) and 30th (4.16 ± 0.75) minutes compared to resting (0.97 ± 0.09). There was no statistical difference between the three conditions (Figure 2). There was no statistical difference between the three conditions and between the 15th and 30th minutes in the three conditions (Figure 2).

**Figure 3:**
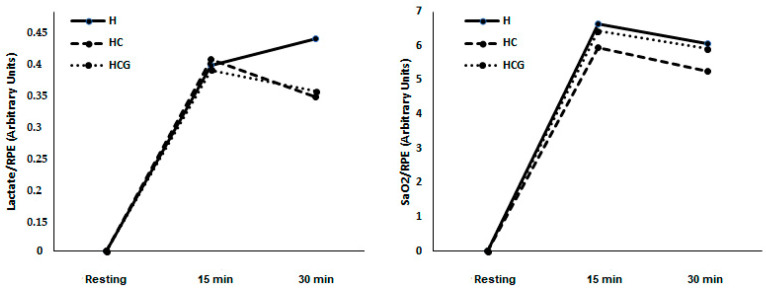
Lactate/RPE ratio and SpO_2_%/RPE ratio demonstrated as a mean. Two-way ANOVA and Tukey’s test were used, *n* = 9 volunteers. For *p* < 0.05. Hypoxia (H), hypoxia + carbohydrate (HC), and hypoxia + carbohydrate + glutamine (HCG).

There was no difference in lactate/RPE and SpO_2_%/RPE ratios in the studied moments and conditions (Figure 3). 

In HC, glucose was higher at the 15th minute compared to resting and at the 30th compared to resting. In HCG, glucose was higher at the 30th minute compared to resting. There is no difference between the conditions or in the glucose/RPE ratio (Table 1).

## 4. Discussion

High altitude hypoxia represents one challenge for exercise physiology and human performance because of early fatigue which compromises performance [24]. In this study, we evaluated whether supplementation with carbohydrate alone and associated with glutamine modifies RPE during exercise at 70% of peak oxygen uptake in hypoxia. Our main result is that glutamine and carbohydrate supplementation does not prevent the high RPE during exercise.

The permanence in hypoxia because of high altitudes can induce several alterations in the human organism, including cardiovascular, immunological, and metabolic [25,26,27]. Performing physical exercise in this condition makes the changes promoted by exercise even more evident [28,29]. On the other hand, strategies such as training at sea level but resting/sleeping at high altitudes can be useful to try to maximize acclimatization and adaptation in training [30].

Although the participants remained at rest for the two hours preceding exercise, the SpO_2_% at the beginning of the exercise was lower than the expected normoxic values for humans, proving the condition of hypoxia indicated in our study, similarly to other studies [31,32]. SpO_2_% decreased even more after 15 min and remained at a lower level after 30 min of exercise without supplementation. The same pattern occurred when the participants consumed supplementation, confirming a previous study [9,33]. However, the effects of carbohydrate and glutamine supplementation on SpO_2_% are still controversial, since other studies have shown an increase in SpO_2_% after supplementation [1,34]. Carbohydrate supplementation can increase the rate of glucose oxidation, increasing CO_2_ production that would stimulate breathing control centers for CO_2_ excretion and increasing SaO_2_ [35]. Glutamine supplementation, on the other hand, could change the central concentration of glutamate, an excitatory neurotransmitter that would increase ventilation, helping to restore SaO_2_ [36]. The effects of supplementation can be limited. Perhaps, the high intensity associated with the time of exposure to hypoxia may represent a breakdown of body homeostasis greater than the capacity of action of carbohydrate and glutamine supplements.

The reduction in SpO_2_% in the three conditions can limit the time of exhaustion since it can be considered one of the leading causes of difficulty in performing any exercise in high altitude conditions. There was no difference in the time of exhaustion in the placebo conditions and when the participants consumed supplementation. These results contradict the literature, because several studies show the effects of carbohydrate supplementation on performance [18,37]. We believe that the impact of the reduction in SpO_2_% has outweighed the beneficial effects of carbohydrate supplementation during exercise in maintaining performance mediated by an increase in blood glucose, as shown in Table 1.

Blood glucose may have played an essential role in controlling RPE during exercise, since glucose was higher at the 30th minute in both supplements compared to the resting value. As shown in Figure 2, RPE increased at the 30th minute compared to the 15th in the hypoxia condition. After carbohydrate supplementation, as well as after carbohydrate and glutamine supplementation, there was no difference between the 15th and 30th. Although there is no difference between the three conditions, these results suggest that both supplements can inhibit the increase in RPE at the 30th minute of exercise. In normoxia, RPE is associated with glycemia and carbohydrate supplementation during long-term exercise [38]. During moderate exercise in hypoxia similar to 4200 m, carbohydrate supplementation can mitigate the increase in RPE [13]. However, this is the first study carried out to evaluate the effects of supplementation during intense exercise in hypoxia and suggest that the effects of supplementation on RPE may depend on blood glucose and not on the intensity of exercise or SaO_2_.

The level of fatigue or tiredness during exercise can be measured using the RPE scale, as it reflects the psychological and physiological perceptions related to performance on a psychobiological scale.

The hypoxic environment worsens psychobiological characteristics, including memory, attention, decision making and learning [4] and mood as intellectual processes, perceptions, and concepts, as well as depression, tension, confusion, fatigue and vigor [5,6] that can affect RPE. 

Glutamine is an amino acid found in greater quantity in the blood or skeletal muscle and can perform numerous functions, including synthesis of neurotransmitters such as glutamate [39]. The supplementation of glutamine over a prolonged time increases glutamine in several tissues [40,41,42]. Previous studies have shown that glutamine supplementation aids cognition and mood [20,43]. For that reason, we believe it is possible that the accumulation of endogenous glutamine, over the six days of supplementation, may have contributed to increasing the central concentration of glutamate, an essential excitatory neutral transmitter, helping to mitigate the increase in RPE found in our study.

The concentration of lactate [44], hemodynamic changes such as changes in HR [45], and SpO_2_% [7,32] could explain the effects of supplementation with carbohydrate and glutamine. However, there was no change in these parameters between the three conditions.

We conclude that previous supplementation with glutamine and carbohydrate during intense exercise in hypoxia similar to 4200 m can attenuate the increase in RPE by the increase in glycemia and can be a useful strategy for people who exercise in these conditions. This study was carried out with nine volunteers, and this may have been a limitation. This study paves the way for new studies aiming to understand how nutrition can act in extreme physiological conditions. However, this conclusion must be viewed with caution, since the differences found in the HCG group were due to time and not compared to the other groups. New studies should be carried out, with a more significant number of participants, at different levels of hypoxia, and with different intensities and durations of exercise.

## Figures and Tables

**Table 1:** Glucose concentration and glucose/RPE ratio.

		Hypoxia	Hypoxia + Carbohydrate	Hypoxia + Carbohydrate + Glutamine
Glucose	Resting	62.20 ± 3.28	58.03 ± 1.80	60.13 ± 2.18
	15 min	73.87 ± 3.86	77.30 ± 4.86 ^a^	68.70 ± 5.15
	30 min	109.45 ± 25.47	86.96 ± 8.36 ^a^	81.15 ± 4.75 ^a^
Glucose/RPE	Resting	-	-	-
	15 min	6.14 ± 0.59	5.85 ± 0.71	5.31 ± 0.46
	30 min	8.07 ± 1.78	5.53 ± 0.47	5.87 ± 0.46

Glucose (mmol) and glucose/RPE ratio demonstrated as a mean. Two-way ANOVA and Tukey’s test were used, *n* = 9 volunteers. ^a^ was different from the resting condition. For *p* < 0.05.
